# Anxiety mediates association between sex and jaw function limitation in temporomandibular disorder patients from China

**DOI:** 10.3389/fneur.2024.1398788

**Published:** 2024-05-13

**Authors:** Li Chen, Shuyuan Zhang, Yanyue Tan, Yunhao Zheng, Shanbao Fang, Yating Yi, Xin Xiong

**Affiliations:** ^1^State Key Laboratory of Oral Disease, National Clinical Research Center for Oral Diseases, West China Hospital of Stomatology, Sichuan University, Chengdu, China; ^2^West China Hospital of Sichuan University, Chengdu, China; ^3^Department of Nursing, West China Hospital of Stomatology, Sichuan University, Chengdu, Sichuan, China; ^4^Department of Orthodontics, College and Hospital of Stomatology, Guangxi Medical University, Nanning, China

**Keywords:** anxiety, sex, jaw function, temporomandibular disorders, mediation analysis, gender equity

## Abstract

**Aim:**

The objective of this study is to explore the relationship between sex and jaw function and to test whether anxiety mediates the causal relationship between sex and jaw function in temporomandibular disorders (TMDs) patients.

**Methods:**

A total of 488 participants with TMD were included in the analysis. Demographic data were collected. Generalized anxiety symptoms and anxiety severity were initially assessed using the GAD-7 questionnaire. And jaw function limitation was measured using the JFLS-8 scale. A directed acyclic graph (DAG) was used in this study to evaluate the hypotheses. Mediation analysis was conducted to explore causality and to calculate the total effect, natural direct effect (NDE) and natural indirect effect (NIE).

**Results:**

In TMD patients, there was a significant association between female and jaw function (*r* = 0.17, *p* < 0.001), female and anxiety (*r* = 0.15, *p* = 0.002), anxiety and jaw function (*r* = 0.35, *p* < 0.001). In addition, sex can directly lead to differences in impaired jaw function (NDE: 3.719, 95% CI: 1.619–5.828, *p* < 0.001), and can also be causally related to jaw function through anxiety (NIE: 1.146, 95% CI: 0.267–2.024, *p* = 0.011). And the total effect was 4.865 (95% CI, 2.709–7.029, *p* < 0.001).

**Conclusion:**

A causal mechanism was found that anxiety acts as a mediator of sex effects on jaw function. Therefore, psychological factors need to be taken into account in the treatment of female TMD patients. Further clinical trials are needed to explore whether psychotherapy is more beneficial to improve jaw function in female TMD patients.

## 1 Introduction

Temporomandibular disorder (TMD) is an umbrella term for a group of disorders, including musculoskeletal and neuromuscular disorders, that share the same or similar pathogenetic factors and clinical symptoms. TMD can affect the temporomandibular joint (TMJ), the masticatory muscles and their associated structures (1). Although TMD is generally self-limiting (2, 3), it can have a serious impact on quality of life, jeopardizing the normal functioning of the oral and maxillofacial system, including clinical symptoms such as orofacial pain, stiffness, limited voice and jaw movements (4, 5). TMD is also associated with different types of primary headache disorders and chronic daily headache (6, 7), and affecting the patient’s facial aesthetics, as well as their physical and psychological health (8–11). When TMJ disorders and primary headache disorders co-exist in comorbidity, diagnosis and treatment become a challenge (7).

Epidemiologic studies have shown that about 50–75% of the population is affected to some degree and shows some signs of TMD. Traditionally, TMD has been thought to affect mainly females of childbearing age (12). There is evidence that the prevalence is approximately two times higher in females than in males (13), and is most common in the second through fourth decade of reproductive life (14). TMD is often thought to be multifactorial, including psychosocial factors. However, the reasons for the higher prevalence of TMD in females than in males remain unclear. Recent research suggests that a combination of genes, psychological and perception of pain may play a role in the onset of TMD as well as its prolonged duration (15, 16).

TMD is considered to be related to psychosomatic factors such as anxiety, stress, and sleep disorders (17, 18). Among them anxiety disorders are the most common of all mental disorders. In 2019, 301 million people worldwide suffer from anxiety disorders, with more females than males suffering from them (19). Affective distress (in form of anxiety) and anxiety sensitivity have been associated with pain in experimental and clinical-based investigations. A bidirectional relationship between TMD and anxiety is thought to exist and multiple psychological variables can serve as predictors of TMD (20). Several studies have pointed to a higher prevalence of anxiety in patients with TMD compared to healthy individuals (16, 21, 22). And psychosocial disorders play an important role in the development of TMD (11, 23).

Most of the current studies have focused on sex differences in the incidence of TMD and pain levels in patients with TMD, while fewer studies have examined whether there are differences in life-affecting jaw function limitations among patients with TMD. The specific association between TMD and anxiety has been the subject of several studies, and there have been a number of studies that have explored the mechanisms behind this through functional MRI (24). At the same time, sex differences in TMD have been consistently observed, but few studies have considered that anxiety may play an effect in the middle of this. To our knowledge, there are currently no studies that have considered anxiety as a mediator to conduct causal analysis between sex and the causal relationship of TMD.

Because of the difficulty of objectively measuring pain and function, the use of patient-reported outcomes (PROs) in dentistry is essential for the evaluation and diagnosis of TMJ disorders and headaches (25, 26). The Jaw Functional Limitations Scale (JFLS) is the International Network for Orofacial Pain and Related Disorders Methodology’s recommended PROM for the evaluation of jaw-related disabilities (25). JFLS is a self-administered questionnaire developed in 2008 that exhibits improved psychometric characteristics and has good validity in TMD patients (27). Compared to the JFLS-20, the JFLS-8 is shorter while demonstrating excellent content validity, construct validity, internal consistency (*α* = 0.82, only TMD patients), and reliability (CCC rho = 0.81) of the JFLS-8 questionnaire (27, 28). Therefore, in this study JFLS-8 was chosen to measure jaw function limitation.

The present study initiated with the aim of exploring the relationship between sex and jaw function and testing the hypotheses that anxiety mediates the causal relationship between sex and jaw function.

## 2 Materials and methods

### 2.1 Participants

This observational cross-sectional study was performed at the Department of Temporomandibular Joint, West China Hospital of Stomatology, Sichuan University, from February 2022 to October 2022. This study was approved by the Ethics Committee of West China Hospital of Stomatology of Sichuan University (WCHSIRB-D-2022-137) and in compliance with the Declaration of Helsinki. The inclusion criteria for this study were: (a) consent to participate in this study, (b) the data of patients was complete and sufficient and (c) diagnosed with TMD by a TMD specialist based on DC/TMD protocols. Exclusion criteria were (a) children/adolescents aged below 18, (b) cognitive impairment or illiteracy and (c) no history of facial surgery or non-operative facial injuries. According to the sample size estimate, a minimum of 119 participants was required for this study at Cohen’s f^2^ = 0.15 (medium effect size), *α* = 0.05, and power = 0.95.

### 2.2 Measurements

Data included demographics, Generalized Anxiety Disorder 7-item (GAD-7) and Jaw Functional Limitation Scale, 8-item version (JFLS-8). Demographics included age, sex, education level and income of the participants. The age of the participants was recorded as a continuous variable in years, and the sex variable was recorded on the basis of the sex registered in the ID card (29). Education levels were categorized into three groups: (1) elementary/junior high/senior high school degree, (2) college/bachelor’s degree, and (3) master’s/doctor’s degree. We categorized the *per capita* household income of the participants into three categories: (1) less than 3,000 RMB per month; (2) 3,000–6,000 RMB per month; and (3) more than 6,000 RMB per month.

The GAD-7 questionnaire was used to initially assess generalized anxiety symptoms and the severity of anxiety. The respondents were supposed to select a description that matches their behavior and feelings over the past 2 weeks on a scale ranging from 0 (not at all) to 3 (nearly every day). As recommended by the latest Diagnostic Criteria for Temporomandibular Disorders (DC/TMD) (30) diagnostic criteria, the JFLS was used to measure function limitation in patients with TMD (30, 31). The jaw function limitation in this study was measured with the 8-item version. This 8-item instrument were developed as a global measure of functional limitation with each item scoring from 0 (no limitation) to 10 (severe limitation) for different levels of limitation in jaw activities including chewing, smiling, swallowing, etc.

### 2.3 Statistical analysis

**Figure 1:**
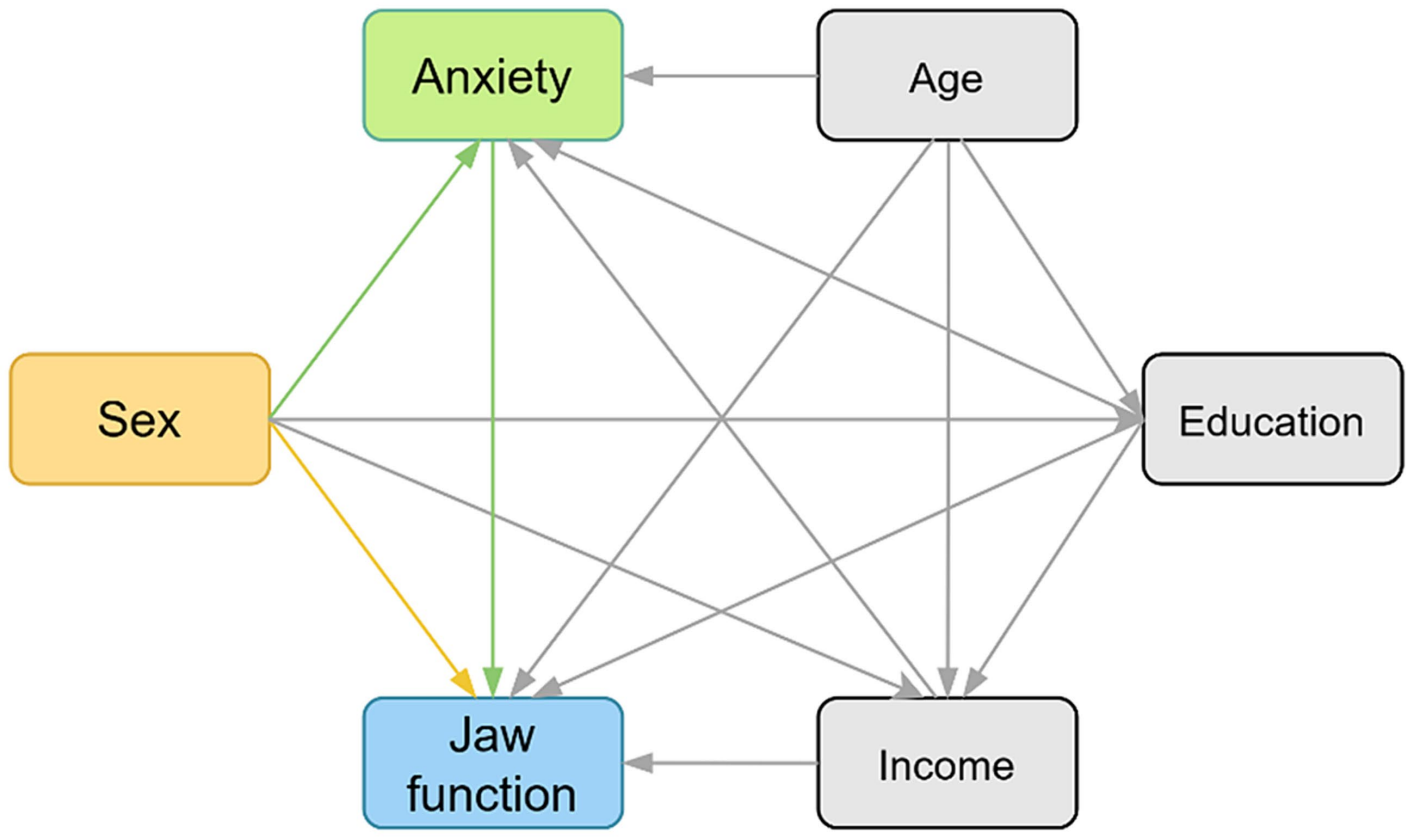
The hypothesized relationships between variables and jaw function demonstrating by directed acyclic graph (DAG). The yellow box indicates exposure. The green box indicates mediator. The blue box indicates outcome, and the gray boxes indicate confounders. The yellow path indicates the direct effect and the green paths indicate the indirect effects.

A directed acyclic graph (DAG) was used in this study to evaluate the hypotheses of the study based on *a priori* knowledge (Figure 1). The primary endpoint (dependent variable) was the JFLS-8 score. Sex was considered as an independent variable according to previous studies 122 because of their potential impact on mandibular functional limitations. The remaining demographic characteristics (age, education, income) were analyzed as covariates due to their no effect on sex but on the measured outcome (JFLS-8 score).

The Shapiro–Wilk test was employed to test the normality of the variables. The differences between categorical variables were compared using the chi-square test and ordinal variables using the Mann–Whitney U test. To initially explore the relationship between the variables, the Spearman correlation coefficients were calculated and adjusted by the Benjamin-Hochberg method.

Causal mediation analysis is conducted to explore causal relationships and obtain direct and indirect effect values. This analysis decomposed the total effect of exposure into estimates of natural direct and indirect effects. Indirect effects are the effects through mediators in the total effect of exposure on the outcome, whereas direct effects reflect the remainder of the total effect. The 95% confidence intervals (CI) of the estimates were calculated using the Bootstrap method (5,000 stimulations).

**Figure 2:**
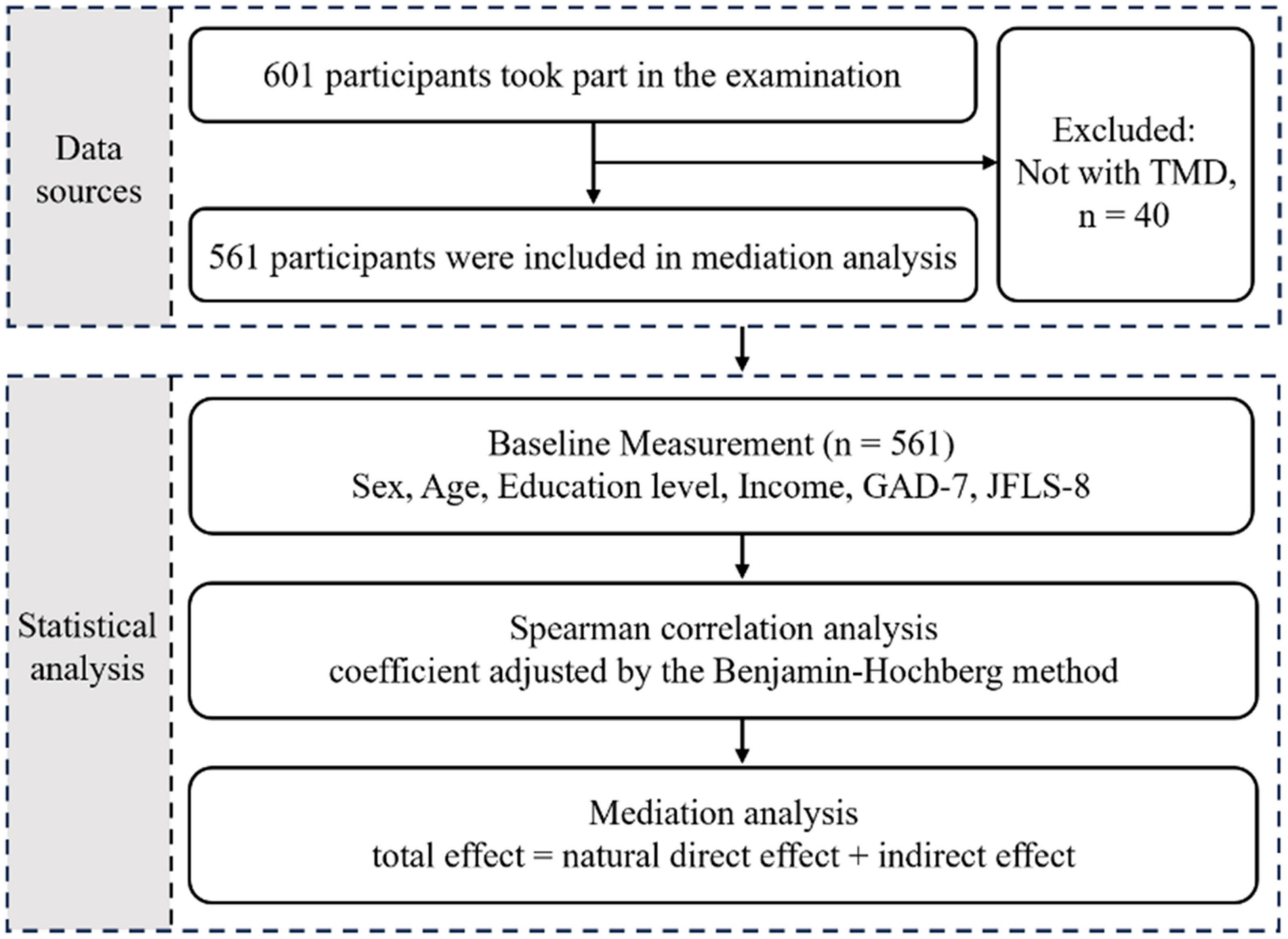
Overview of study design and workflow. GAD-7, Generalized Anxiety Disorder 7-item; JFLS-8, Jaw Functional Limitation Scale, 8-item version.

Analyses were performed using R version 4.3.1 (R Foundation) with a *p* < 0.05 indicating significance. The regression model was generated by the R package ‘modelsummary’ (version 1.2.0). The mediation analysis was conducted through R package “medflex” (version 0.6–10). The R package “likert” (version 1.3.5) was used for likert plot to visualize the results of likert scales including GAD-7 scale and JFLS-8 scale. The workflow is depicted in Figure 2. Above analyses were used and interpreted in compliance with the STROBE (Strengthening the Reporting of Observational Studies in Epidemiology) checklist.

## 3 Results

### 3.1 Baseline characteristics of the study population

Of the 601 individuals who completed the questionnaire, 73 participants younger than 18 years old and 40 individuals without TMD based on the definition of DC/TMD were excluded. A final total of 488 were included in the following analyses, with 99 males and 389 females (Table 1), most of whom were from southwest China. All variables were non-normally distributed (*p* < 0.05). Of the whole study population, the median age was 27 years, and the median JFLS-8 and GAD-7 scores were 13 and 4, respectively. Compared to the median score of 9.5 in the male group, the female group had a significantly higher JFLS-8 score (median 14.0, *p* < 0.001). Similarly, the median GAD-7 score was significantly higher among females (median 4.0, *p* = 0.001) than among males (median 2.0). In addition, the income of the two groups showed a significant difference (*p* = 0.020), while there was no significant difference in age (*p* = 0.134) and education level (*p* = 0.467) between the male and female participants. Based on the sample size included in this study, the effect size was calculated to be 0.035 at *α* = 0.05 and power = 0.95.

**Table 1:** Baseline characteristics of the study population.

Sex	Male	Female	*p*-value	Total
N (%)	99 (%)	389 (%)		488
Age	30.0 (23.0–37.0)	26.0 (23.0–33.0)	0.134	27.0 (23.0–34.0)
JFLS-8	**9.5 (3.0–17.0)**	**14.0 (6.0–22.0)**	**<0.001**	13.0 (5.0–21.0)
GAD-7	**2.0 (0.0–6.0)**	**4.0 (1.0–7.0)**	**0.001**	4.0 (1.0–7.0)
Education			0.467	
Elementary/junior high/senior high school degree	22 (22.2%)	66 (17.0%)		88 (18.0%)
College/bachelor’s degree	66 (66.7%)	280 (72.0%)		346 (70.9%)
Master’s/doctor’s degree	11 (11.1%)	43 (11.1%)		54 (11.1%)
Income (RMB per month)			**0.020**	
<3,000	**14 (14.1%)**	**60 (15.4%)**		74 (15.2%)
3,000–6,000	**33 (33.3%)**	**183 (47.0%)**		216 (44.3%)
>6,000	**52 (52.5%)**	**146 (37.5%)**		198 (40.6%)

The differences between groups were compared by chi-square test or Mann–Whitney U test. (*p*-value < 0.05 indicating significance are shown in bold). Values are displayed as median (Q1–Q3) or n (%). JFLS-8, the total score of the JLFS-8; GAD-7, the total score of the GAD-7. GAD-7, Generalized Anxiety Disorder 7-item; JFLS-8, Jaw Functional Limitation Scale, 8-item version.

**Figure 3:**
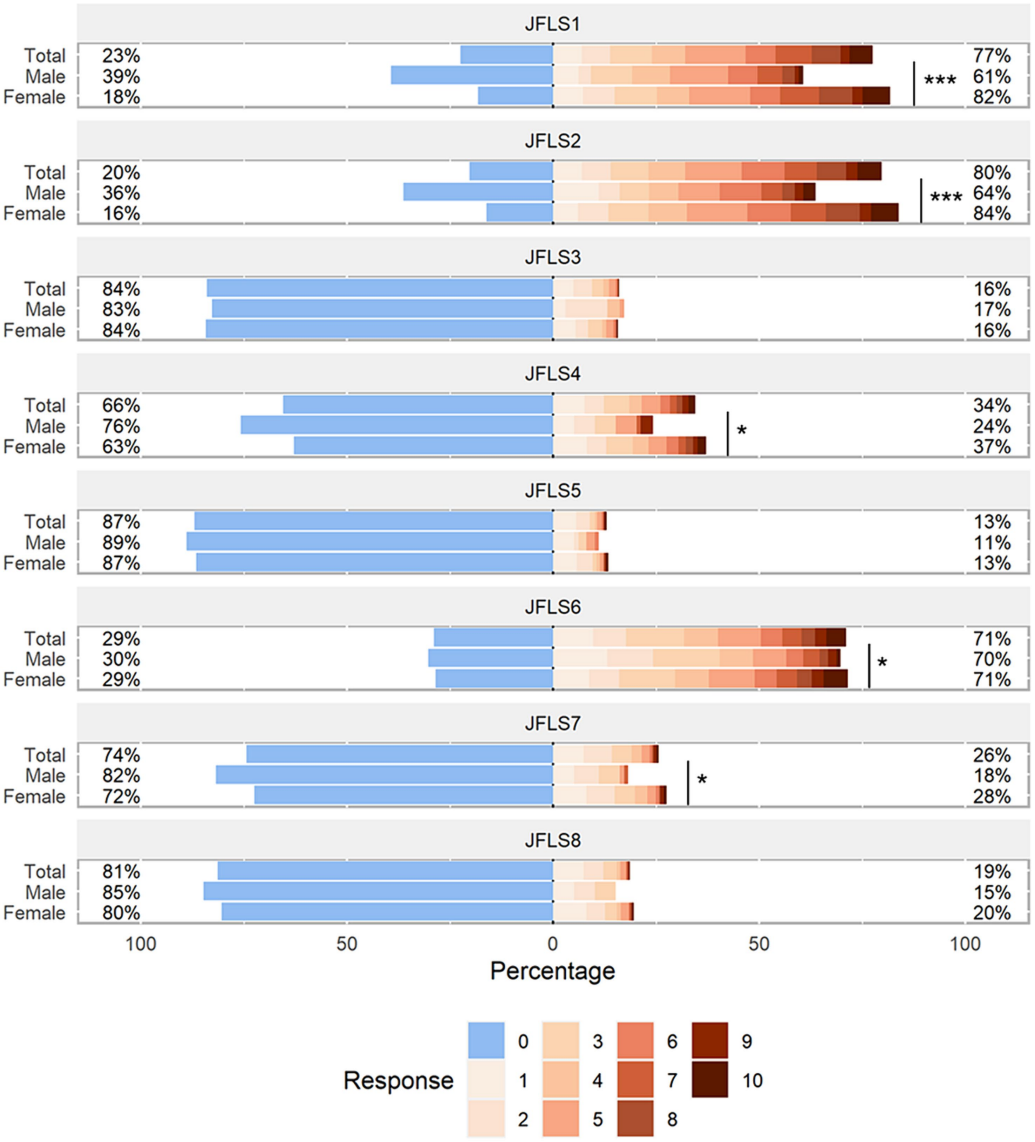
Distribution of JFLS-8 scale responses. The likert plot was used to graphically represent the mean values of each item scores of JFLS-8 among males and females. *P*-values were calculated by the Mann–Whitney U test. The number after the JFLS indicates the item number in the JFLS questionnaire. JFLS, Jaw Functional Limitation Scale. *p < 0.05; **p < 0.01; ***p < 0.001.

**Figure 4:**
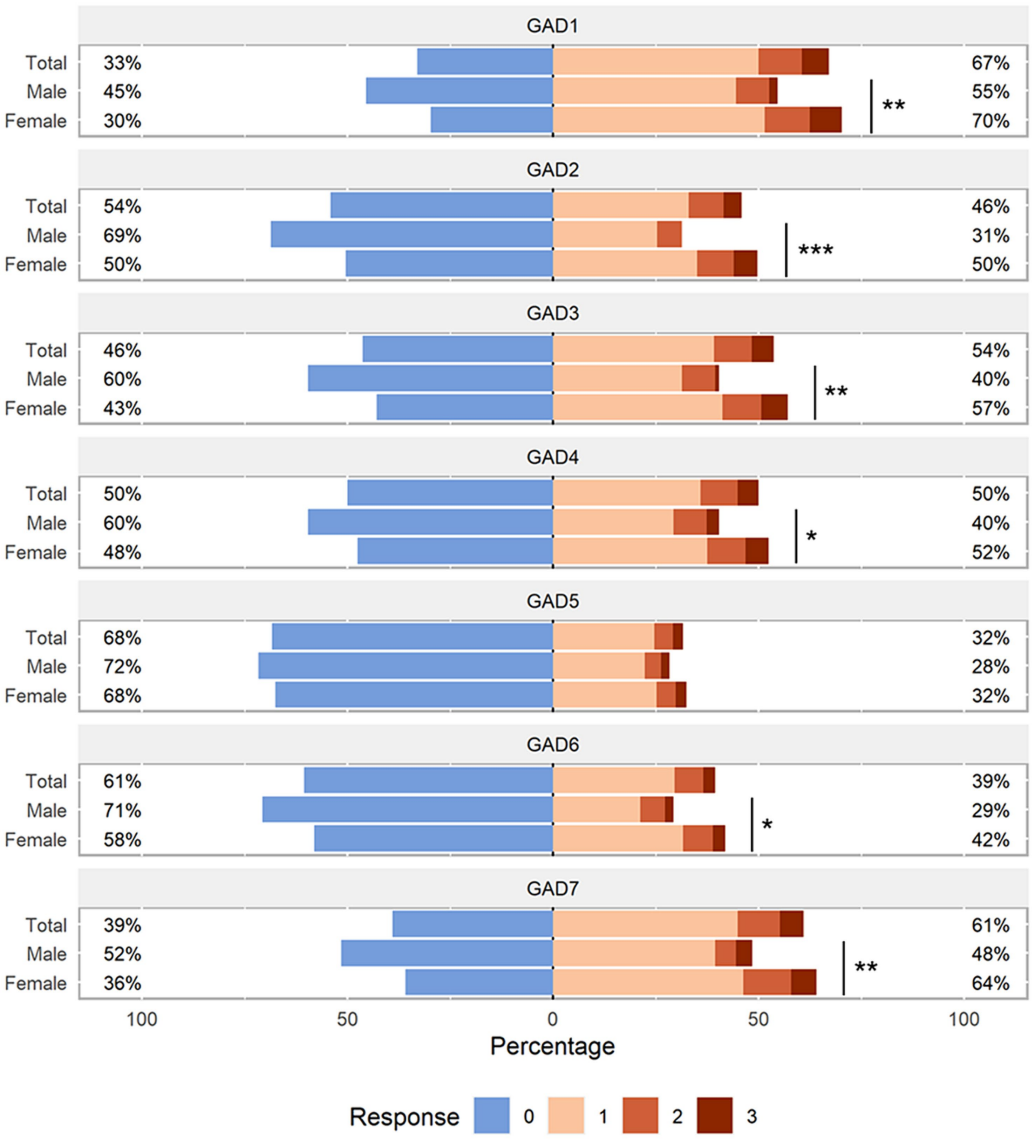
Distribution of GAD-7 scale responses. Likert plot was used to graphically represent the participants’ response to each item of the GAD-7 questionnaire stratified by sex. *P*-values were calculated by the Mann–Whitney U test. The number after the GAD indicates the item number in the GAD questionnaire. GAD, Generalized Anxiety Disorder. *p < 0.05; **p < 0.01; ***p < 0.001.

The JFLS-8 scale and GAD-7 scale used were in good consistency, and their reliability as assessed by the Cronbach’s alpha coefficient was 0.800 (95% CI: 0.773–0.826) and 0.932 (95% CI: 0.922–0.941) respectively. The responses of JFLS-8 and GAD-7 were visualized in Figures 3, 4. The results showed that the median scores on chewing tough food (males: 3.0, females: 4.0, *p* < 0.001) and chewing chicken (males: 2.0, females: 5.0, *p* < 0.001) were significantly higher in females than in males. Similar results were observed in the median scores of opening wide enough to drink from a cup (males: 0.0, females: 0.0, *p* = 0.015), yawning (males: 2.0, females: 3.0, *p* = 0.047) and talking (males: 0.0, females: 0.0, *p* = 0.050). As for the GAD-7 scale, the median scores were significantly higher in females than in males on all items except item 5.

### 3.2 Correlation between variables

Spearman’s correlation coefficients adjusted with the Benjamini-Hochberg method are shown in Table 2. It revealed that JFLS-8 scores were significantly and positively correlated with female gender (*r* = 0.17, *p* < 0.001) and GAD-7 scores (*r* = 0.35, *p* < 0.001), where female in turn was significantly and positively correlated with GAD-7 scores (*r* = 0.15, *p* = 0.002). Additionally, significant negatively correlations were also observed between JFLS-8 scores and income (*r* = −0.17, *p* < 0.001). Meanwhile, female is negatively correlated with income (*r* = −0.10, *p* = 0.051), and although it does not achieve significant level, the *p* value is very close to 0.05.

**Table 2:** Spearman correlation coefficient matrix.

	Sex	Age	Education	Income	GAD-7
Age	−0.07				
Education	0.04	−0.25***			
Income	−0.10	0.01	0.29***		
GAD-7	0.15**	−0.05	−0.08	−0.06	
JFLS-8	0.17***	0.03	−0.08	−0.17**	0.35***

The *p*-values are adjusted with the Benjamini–Hochberg method. GAD-7, Generalized Anxiety Disorder 7-item; JFLS-8, Jaw Functional Limitation Scale, 8-item version. **p < 0.01, ***p < 0.001.

**Table 3:** Multiple regression models for the association between variables and jaw function.

	Estimate	95% CI	Estimate	95% CI
Age	0.100*	(0.001, 0.199)	0.106*	(0.006, 0.205)
Sex				
Male	Reference	Reference	Reference	Reference
Female	3.726**	(1.292, 6.161)	4.025**	(1.592, 6.457)
Education				
Elementary/junior high/senior high school degree	Reference	Reference	Reference	Reference
College/bachelor’s degree	−0.506	(−3.379, 2.367)	−1.202	(−4.027, 1.623)
Master’s/doctor’s degree	−0.686	(−4.685,3.313)	−1.796	(−5.702, 2.109)
GAD-7	0.761***	(0.552, 0.969)	0.780***	(0.572, 0.988)
Income	−1.710*	(−3.154, −0.266)		

CI, confidence interval. *p < 0.05, **p < 0.01, ***p < 0.001.

### 3.3 Multiple regression models

Multiple regression models (Table 3) showed that JFLS-8 score was significantly positively associated with GAD-7 score (0.761, 95% CI: 0.552–0.969, *p* < 0.001) after adjustment for age, sex, education and income. In addition, JFLS-8 score was also significantly positively associated with age (0.100, 95% CI: 0.001–0.199, *p* = 0.048) and female (3.726, 95% CI: 1.292–6.161, *p* = 0.003), while JFLS-8 score was negatively associated with income (−1.710, 95% CI: −3.154 – −0.266, *p* = 0.020). After adjustment for age, sex and education (without including income), JFLS-8 score was significantly positively associated with age (0.106, 95% CI: 0.006–0.205, *p* = 0.037), female (4.025, 95% CI: 1.592–6.457, *p* = 0.001) and GAD-7 score (0.780, 95% CI: 0.572–0.988, *p* < 0.001).

### 3.4 The mediating relationship of GAD-7 scores between sex and JFLS-8 scores

**Figure 5:**
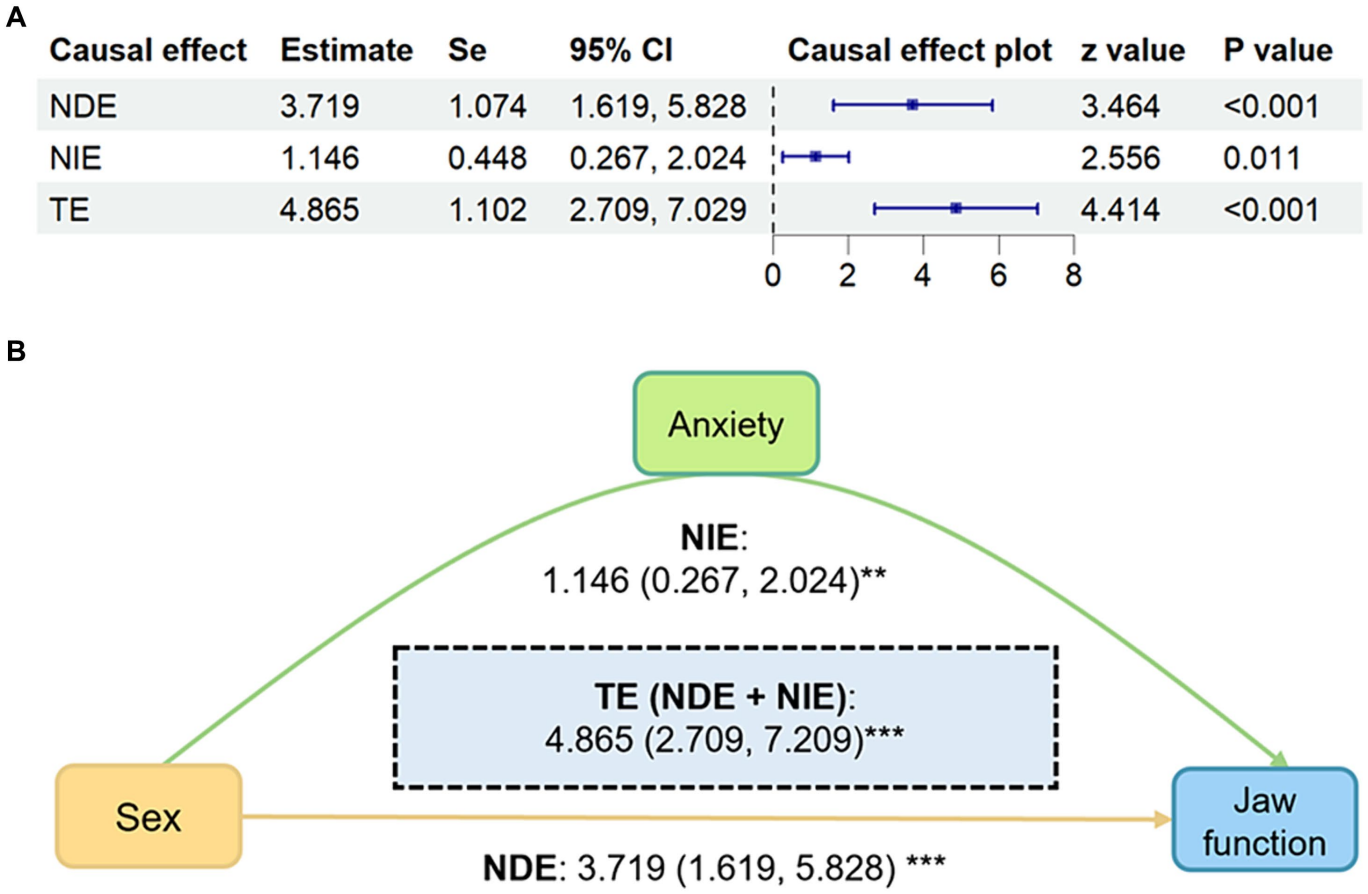
Mediation analysis results of sex predicting JFLS-8 mediated by GAD-7. **(A)** Forest plot of mediation analysis results. **(B)** Causal directed acyclic graph of mediation analysis results shows the mediation analysis of GAD-7 scores (anxiety) on the relationship between sex and JFLS-8 (jaw function). The yellow path indicates the direct effect and the green paths indicate the indirect effects. Estimates and CIs calculated using 5,000 bootstraps. All models adjusting for age, education and income. Values are displayed as β (95%CI). CI, confidence interval; NDE, natural direct effect; NIE, natural indirect effect; TE, total effect. *p < 0.05; **p < 0.01; ***p < 0.001.

On the basis of the DAG, sex and GAD-7 scores were selected to explore whether sex had a causal effect on JFLS-8 and whether GAD-7 had a mediating effect therein. Mediation analysis showed that sex had a significant causal effect on both jaw function and anxiety (Figure 5). Meanwhile, anxiety played a mediator role in the pathway of female was more likely to lead to jaw function limitation. The total effect of sex on JFLS-8 scores was 4.865 (NDE: 95% CI: 2.709–7.029, *p* < 0.001). GAD-7 scores mediated 22% of the total causal effect (NIE: 1.074, 95% CI: 0.267–2.024, *p* = 0.011). After adjusting for the mediating effect of GAD-7 scores, the direct effect (sex on JFLS-8 scores not due to GAD-7 scores) was 3.719 (95% CI: 1.619–5.828, *p* < 0.001).

## 4 Discussion

In the present study, we found that in TMD patients there was a significant causally association between female and jaw function, female and anxiety, anxiety and jaw function. Moreover, a causal mechanism was observed that sex can directly affects jaw function, and can also limit jaw function by causing anxiety.

Our study reports a predominance of females among TMD patients, which is consistent with previous studies (12, 32). A recent systematic review and meta-analysis (13) found that females are twice as likely as males to develop TMD. Sex has long been medically defined as a biological trait, and the primary sex determination in humans occurs at fertilization and is dependent on the two sex chromosomes of the fertilized egg (33). Interventions to change a person’s external sex characteristics, including the use of sex hormones and other medications, do not change a person’s genetic code and cannot alter biological sex (34). Therefore, sex can be considered one of the causes of TMD based solely on its correlation with TMD without the need for a causal analysis. Although it is not entirely clear what aspects of biology, psychology, or social roles are responsible for females being more likely to suffer from TMD than males, various explanations have been put forward (35). There is the notion that females are more willing to report pain (36, 37) and attend clinics than males, and that the mechanism behind this may be related to sex differences in pain thresholds and tolerance times due to ovarian hormones (38–40).

The results showed the total and direct effects of sex on jaw function and the mediating effect of anxiety were significant. Our study confirms a causal mechanism of sex differences in jaw function limitation that sex induces differences in the anxiety level, and consequently impacts jaw function. There is a bidirectional relationship between anxiety disorders and TMD (41, 42). The prevalence of anxiety is higher in patients with TMD (21, 22). Some studies have speculated that the prevalence of TMD may be associated with the fact that women are under greater stress in society (43, 44). Anxiety has been shown to affect muscle activity (45, 46) and may also affect changes in the bones of TMJ (46, 47). Anxiety may affect metabolism and blood flow by altering sympathetic nervous system (48, 49), e.g., reduced muscle blood perfusion; reduced proprioceptive inputs, etc. directly affecting the performance of the masticatory muscles, thereby reducing the efficiency of complex movements such as chewing, leading to increased fatigue and ultimately reduced jaw function.

The mechanism underlying this may be related to anterior cingulate cortex and anterior insula. They are two important regions responsible for encoding the emotional and motivational aspects of pain. They have been studied by functional and structural magnetic resonance imaging (MRI) methods and are currently thought to be associated with orofacial pain (50). A joint analysis of mastication and orofacial pain studies showed joint activation of the left insula, left primary motor cortex, and right primary somatosensory cortex (51, 52). In a longitudinal treatment study, activation in the right anterior insula and right cerebellum was reduced in patients with TMD during treatment (53). And anxiety-related TMD symptoms may be associated with the anterior insula. It has been shown that a reduction in anxiety in TMD patients during splinting is associated with a reduction in functional magnetic resonance imaging activation in the left anterior insula and right posterior insula (24). This may be related to the fact that the anterior insula is interconnected with the limbic system (amygdala, anterior prefrontal, and cingulate cortex) thus the anterior insula acts as an important pivot between the internal emotional state and somatosensory inputs to somatosensory cell labeling (54).

A large number of patients with TMD are suffering from a sex-differentiated decrease in life satisfaction (11). Psychological factors need to be taken into account in the treatment of female TMD patients due to the result that 22% of the total effect of sex on the development of TMD was mediated by anxiety. This could mean that the assessment and interventions of anxiety are considered to have an impact on the improvement of jaw function limitation. Previous studies have revealed that both psychological and oral interventions are beneficial in the relief of anxiety or TMD symptoms (24, 55, 56). Patients with TMD improved significantly when provided with treatments to get anxiety under control, including medication (57, 58), stress management (59), and biofeedback (60, 61). Our study suggested that treatment for anxiety may be more beneficial in improving jaw function in female TMD patients compared to male patients and future clinical trials are needed to explore whether psychotherapy is more effective for female TMD patients and to elucidate the biological mechanisms involved.

A number of considerations should be taken into account when interpreting the results. The main limitation is that the severity of anxiety and jaw function limitation was assessed by patients completing a self-assessment questionnaire without a professional diagnosis by a relevant medical professional. This may lead to a less accurate diagnosis and assessment. Secondly, the demographic information is incompletely considered at the time of collection. Estimates of direct and indirect effects will be affected if there is an uncontrolled common cause of the mediator and the outcome affected by the exposure. However, the major factors associated with exposure and outcome have been taken into account. Third, the questionnaire collected in this study was for anxiety but not for stress, which may also be mediators of sex-induced jaw function limitation. Similarly, income may also be a mediator. Thus, future studies could analyze the causal effect between them and sex and jaw function. Moreover, we recruited our sample from West China Hospital of Stomatology, thus limiting the generalizability of our findings to local TMD patients since it was only conducted at one study center, and whether the results can be extended to the whole population needs to be further investigated.

## 5 Conclusion

There were significant sex differences in jaw function limitation and the causal mediation analysis revealed that anxiety mediated the effect of sex on mandibular functional limitation in TMD patients. The findings suggest that actions to promote gender equality are needed to ensure equitable population health. Further clinical are needed to determine whether interventions to relieve anxiety may be more effective in improving female TMD patients’ symptoms.

## Data availability statement

The data supporting the findings of this study will be made available by the corresponding author upon reasonable request.

## Ethics statement

The studies involving humans were approved by Ethics Committee of West China Hospital of Stomatology of Sichuan University. The studies were conducted in accordance with the local legislation and institutional requirements. The participants provided their written informed consent to participate in this study. Written informed consent was obtained from the individual(s) for the publication of any potentially identifiable images or data included in this article.

## Author contributions

LC: Methodology, Software, Validation, Visualization, Writing – original draft. SZ: Data curation, Validation, Writing – original draft. YT: Investigation, Visualization, Writing – original draft. YZ: Formal analysis, Methodology, Visualization, Writing – original draft. SF: Formal analysis, Methodology, Validation, Writing – original draft. YY: Validation, Writing – original draft. XX: Conceptualization, Funding acquisition, Project administration, Resources, Supervision, Writing – review & editing.
